# Platelet Dynamics during Natural and Pharmacologically Induced Torpor and Forced Hypothermia

**DOI:** 10.1371/journal.pone.0093218

**Published:** 2014-04-10

**Authors:** Edwin L. de Vrij, Pieter C. Vogelaar, Maaike Goris, Martin C. Houwertjes, Annika Herwig, George J. Dugbartey, Ate S. Boerema, Arjen M. Strijkstra, Hjalmar R. Bouma, Robert H. Henning

**Affiliations:** 1 Department of Clinical Pharmacy and Pharmacology, University of Groningen, University Medical Center Groningen, Groningen, The Netherlands; 2 Sulfateq BV, Groningen, the Netherlands; 3 Department of Anesthesiology, University of Groningen, University Medical Center Groningen, Groningen, the Netherlands; 4 Zoological Institute, University of Hamburg, Hamburg, Germany; 5 Department of Chronobiology, University of Groningen, Center for Behaviour & Neurosciences, Groningen, The Netherlands; 6 Department of Molecular Neurobiology, University of Groningen, Center for Behavior & Neurosciences, Groningen, The Netherlands; 7 Department of Nuclear Medicine & Molecular Imaging, University of Groningen, University Medical Center Groningen, Groningen, the Netherlands; 8 Department of Rheumatology and Clinical Immunology, University of Groningen, University Medical Center Groningen, Groningen, the Netherlands; King’s College London School of Medicine, United Kingdom

## Abstract

Hibernation is an energy-conserving behavior in winter characterized by two phases: torpor and arousal. During torpor, markedly reduced metabolic activity results in inactivity and decreased body temperature. Arousal periods intersperse the torpor bouts and feature increased metabolism and euthermic body temperature. Alterations in physiological parameters, such as suppression of hemostasis, are thought to allow hibernators to survive periods of torpor and arousal without organ injury. While the state of torpor is potentially procoagulant, due to low blood flow, increased viscosity, immobility, hypoxia, and low body temperature, organ injury due to thromboembolism is absent. To investigate platelet dynamics during hibernation, we measured platelet count and function during and after natural torpor, pharmacologically induced torpor and forced hypothermia. Splenectomies were performed to unravel potential storage sites of platelets during torpor. Here we show that decreasing body temperature drives thrombocytopenia during torpor in hamster with maintained functionality of circulating platelets. Interestingly, hamster platelets during torpor do not express P-selectin, but expression is induced by treatment with ADP. Platelet count rapidly restores during arousal and rewarming. Platelet dynamics in hibernation are not affected by splenectomy before or during torpor. Reversible thrombocytopenia was also induced by forced hypothermia in both hibernating (hamster) and non-hibernating (rat and mouse) species without changing platelet function. Pharmacological torpor induced by injection of 5′-AMP in mice did not induce thrombocytopenia, possibly because 5′-AMP inhibits platelet function. The rapidness of changes in the numbers of circulating platelets, as well as marginal changes in immature platelet fractions upon arousal, strongly suggest that storage-and-release underlies the reversible thrombocytopenia during natural torpor. Possibly, margination of platelets, dependent on intrinsic platelet functionality, governs clearance of circulating platelets during torpor.

## Introduction

Hibernation is an energy conserving behavior in animals during winter that is characterized by two phases: torpor and arousal. During torpor, metabolic activity is markedly reduced resulting in inactivity and a drop in body temperature, meanwhile various physiological parameters change including a steep decline in heart rate and ventilation rate [1]–[5]. Bouts of torpor are interspersed by short arousal periods, during which metabolism increases and body temperature returns to euthermia [2], [6], [7]. Key changes in physiological parameters are thought to lead to an increased resistance to ischemia/reperfusion [8], [9] allowing hibernating mammals to survive periods of torpor and arousal without signs of organ injury. Therefore, hibernating animals have been used in various studies as a model to investigate the effects of low body temperature and hypoxia on organs, in attempts to unravel the adaptations that allow these animals to cope with the physiological extreme conditions of torpor [5]. These studies mainly focused on identifying mechanisms employed by these animals to protect their internal organs from injury during hypothermia and rewarming [10]–[14]. The torpid phase embodies several potentially procoagulant conditions, including low blood flow [15], increased blood viscosity [16], [17], immobility, chronic hypoxia, and low body temperature [5]. Although low body temperature has not been described by Virchow in his “triad of risk factors for thrombosis”, it is well established that low temperature leads to platelet activation and aggregation in mammals [18]–[20]. In addition to aggregation, platelet activation also leads to inflammatory reactions and potential organ injury, e.g. via platelet-leukocyte complex formation [21]. Although aggregation of platelets generally lead to thrombus formation, organ injury resulting from thrombotic complications has not been observed in hibernating animals during torpor [5]. We speculate that suppression of hemostasis, as observed by a hypocoagulative state in the 13-lined ground squirrel (*Ictidomys tridecemlineatus*) [22], might play an important role in the prevention of organ injury as well.

Circulating platelet numbers are decreased during torpor in hibernating ground squirrels as compared to summer euthermic animals [23]. Consequently, the blood clotting is reduced during torpor [22], [24]. Upon arousal, platelet numbers are rapidly restored, i.e. within 2 hours upon rewarming to 37°C in ground squirrels [22], [25], [26] and its coagulative function returns to normal [22]. This rapid restoration of platelet count and coagulative function is unlikely to be due to increased platelet production from the bone marrow, because platelet synthesis from megakaryocytes takes 24–48 hours to restore circulating platelet counts after an induced thrombocytopenia [22], [27]. Therefore, the rapid dynamic of restoration of platelet numbers upon arousal suggests a storage-and-release mechanism to underlie thrombocytopenia during torpor rather than clearance-and-reproduction. However, to date, the mechanism(s) that underlie thrombocytopenia during torpor and the full restoration during early arousal are still unclear.

Similarly to platelets, specific classes of leukocytes also disappear from the circulation during torpor [23]. We previously showed the importance of the decrease in body temperature in the mechanism governing the decline in leukocytes, which constitutes of a temperature driven drop in plasma S1P levels [28]. Thus, we hypothesized that body temperature is critical in the initiation of a decrease in circulating platelets. To examine this, we investigated changes in the number of circulating platelets in different stages of natural hibernation in hamster species that undergo either deep multiday torpor bouts or shallow daily torpor. Effects were compared with those found in hamsters, rats and mice that were cooled under anesthesia or in which torpor was induced pharmacologically by 5′-AMP. In order to examine the origin of platelet number decrease and restoration, splenectomy was performed and immature platelet fraction determined. To investigate the coagulative function of the remaining circulating platelets, we performed platelet function measurements by aggregometry and by measurement of platelet activation marker expression by flow cytometry analysis.

Understanding the mechanism of thrombocytopenia and the effect on platelet function in torpor and its subsequent restoration in arousal might lead to new insights to inhibit platelet function or extend platelet shelf life, e.g. under hypothermic conditions.

## Materials and Methods

### Ethics Statement

All animal work has been conducted according to relevant national and international guidelines, and was approved by the Institutional Animal Ethical Committees of the University Medical Center Groningen and University of Aberdeen.

### Hibernation

Prior to experiments, hamsters were kept at summer conditions (L:D cycle of 12 h:12 h) and fed *ad libitum* using standard animal lab chow. To induce hibernation in Syrian hamsters (*Mesocricetus auratus*), the light:dark (L:D) cycle was shortened to 8 h:16 h for ∼10 wk followed by continuous dim light (<5 lux) at an ambient temperature of 5°C. Movement detectors connected to a computer were used to determine the animals’ hibernation pattern. In the Djungarian hamsters (*Phodophus sungorus*), hibernation was induced by shortening the L:D cycle to 8 h:16 h for ∼14 wk at an ambient temperature of 21±1°C. Daily torpor was determined by observation in the middle of the light phase (usual torpor phase) and a single body temperature measurement at the time of euthanization. Animals were sampled related to the time of entry into torpor (at lights on; t = 0 h). Blood was collected from animals at 4 h (torpor), 8 h (arousal) and normothermic animals at 12 h. Blood was collected by cardiac puncture and body temperature was measured i.p. just prior to euthanization.

### Forced Hypothermia

Summer-euthermic Syrian hamsters, Wistar rats, and C57Bl/6 mice were housed at an L:D cycle of 12 h:12 h. The Syrian hamster and Wistar rat were anesthetized by injecting 200 mg/kg ketamine and 1.5 mg/kg diazepam i.p. C57Bl/6 mice were anesthetized by brief isoflurane 2.5% inhalation before ketamine infusion in the jugular vein of 7 mg/hr. Prior to experiments, animals were fed *ad libitum* using standard animal lab chow. Spontaneously breathing hamsters were cooled and rewarmed. In contrast to the hamsters, rats and mice had to be intubated and ventilated to maintain adequate oxygenation. Animals were cooled by applying ice-cold water to their fur and were rewarmed using a water-based or electrical heating mattress and evaporation by airflow. Procedures were adjusted to change body temperature at a rate of ∼1°C per 3 min. Upon reaching 20 degrees body temperature (mouse), 15 degrees (rat), or 8 degrees (hamster), application of ice-cold water was reduced to sustain a stable body temperature for 3 hours in rat and hamster, and for 1 hour in mouse. In the hamster, a catheter was inserted into the jugular vein for blood sampling, while in the rat and mouse a catheter was inserted into the carotid artery to monitor heart rate, blood pressure and draw blood. In hamster, samples were taken on the cooling and rewarming curve. Hence body temperature reflects the time of sampling; e.g. a body temperature of 30°C (coming from 37°C) was reached 3×7 = 21 min after start of cooling. Forced-cooled rats and mice were sampled during euthermia while under anesthesia, 3 hours after cooling the rat and 1 hour after cooling the mouse, and after reaching 37 degrees body temperature upon rewarming. Due to low sample volume, mice were either sampled 1 hour after cooling or after reaching 37 degrees body temperature. Rectal temperature was measured continuously, and heart rate (ECG) was monitored (Cardiocap S/5, Datex Ohmeda).

### Pharmacological Induction of Torpor

C57Bl/6 mice were housed under standard L:D-conditions (L:D cycle of 12 h:12 h) in the animal facilities of the University of Groningen, The Netherlands. Prior to experiments, animals were fed *ad libitum* using standard animal lab chow. Torpor was induced pharmacologically by injecting 7.5 mmol/kg of 5′-AMP (Sigma Aldrich) in 0.9% saline (pH 7.2–7.5) intra-peritoneally. To record body temperature during experiments, we measured the body temperature using a rectal probe (Physitemp Instruments). Mice were euthanized at different times after injection of 5′-AMP or saline. The minimum body temperature during torpor was reached at 4–5 hours following 5′-AMP injection and full arousal with normalization of body temperature occurred by 10 hours after 5′-AMP administration. At euthanization, animals were anesthetized using 3% isoflurane/oxygen and up to ∼800 μl blood was drawn immediately by abdominal aortic puncture into 3.2% sodium citrate and small EDTA-coated tubes. Automated hematological analysis was performed within 5 hours using a Sysmex XE-2100 [29]. The platelets were discriminated from other cells by Forward and Sideward Scatter characteristics. Mature and immature platelets were separated on the basis of Side Scatter, by virtue of the increased amount of granular (i.e. scattering) organelles in immature platelets.

### Splenectomies

Splenectomies were performed on summer-euthermic and torpid Syrian hamsters. Immediately after induction of anesthesia (2–2.5% isofluorane/O_2_), a blood sample was drawn by cardiac puncture, and 4 mg/kg flunixin-meglumin (Finadyne; Schering-Plough) was given s.c. as analgesic. The abdomen was shaved and disinfected by chlorhexidine. The abdominal cavity was opened by a midline incision and the spleen was exposed by careful manipulation of the internal organs using a pair of blunt tweezers. Next, the splenic artery and vein were ligated and the spleen was removed. The abdominal cavity was closed in two layers using ligations. Summer-euthermic animals that underwent splenectomy recovered in a warm room (L:D cycle 8 h:16 h). Once animals started to hibernate, animals were sacrificed during their third torpor bout, which was 60.3±8.1 d following splenectomy. Torpid animals underwent splenectomy during their third torpor bout while being kept at <10°C body temperature using ice-packs. Subsequently, they were allowed to recovered at an ambient temperature of 5°C during which period all animals developed surgery induced arousal. Animals were euthanized upon reaching euthermia.

### Platelet Preparation for Platelet Function Measurements

Rodent blood samples were drawn into 3.2% sodium citrate tubes and stored at room temperature under gentle continuous rotation after being used for flow cytometry preparation. Within 24 hours, platelets were prepared as previously described [30] with small adaptations. Rat blood was centrifuged for 8 minutes at 180×*g* while mouse blood was centrifuged for 11 minutes at 100×*g*. Platelets were then resuspended in buffer A (6 mM dextrose, 3 mM KCl, 0.81 mM KH_2_PO_4_, 9 mM MgCl_2_, 130 mM NaCl, 9 mM NaHCO_3_, 10 mM sodium citrate, 10 mM tris (hydroxymethyl)aminomethane, pH 7.4) as previously described [31] and platelet concentrations were determined on a Horiba ABX Micros 45 hematology analyzer. If needed, platelet suspensions were further diluted in buffer A in order to match with the lowest platelet yield among all samples on that day. These platelet suspensions were then allowed to rest for at least 15 minutes.

### Microtiter Plate Platelet Aggregation (MTP)

Platelet aggregation was determined as previously described [31]. Aliquots (90 μL) of platelet suspension were dispensed on a clear flat bottom 96-wells plate and baseline optical density was measured on BioTek ELx808 absorbance microplate reader every minute. After 6 minutes, 10 μL of ADP and CaCl_2_ in buffer A was added to each well to final concentrations of 20 μM and 1,8 mM respectively. During the remaining 12 minutes run time, the plate was vigorously shaken, not stirred, in between measurements. Separate experiments were corrected by subtraction of baseline absorption. Finally, platelet aggregation was normalized by dividing by the optical densities of an internal standard included in each experiment. To display platelet aggregation, data were transformed to show the increase in light transmission instead of a decrease in optical density.

### Flow Cytometry Analysis for P-selectin

Expression of P-selectin (CD62P), as platelet activation marker, and platelet glycoprotein IIIa (integrin β3 or CD61), as platelet marker, on platelets from rat and mouse whole blood samples was analyzed by double label flow cytometry. In hamster, only the P-selectin antibody could be used. One microliter of whole blood was 1∶25 diluted in phosphate buffered saline (PBS), and incubated with anti-CD61-FITC and/or anti-CD62P-PE with or without 10 uM ADP platelet agonist for 30 min in the dark. The activation was stopped by addition of PBS and fixation by 2% formaldehyde in 300 uL end volume. Samples were stored at 4 degrees in the dark until measurement the next day. Samples were acquired with low flow rate on a FACS Calibur flow cytometer equipped with CellQuest software (BD Biosciences). Samples were analyzed using Kaluza 1.2 software (Beckman Coulter). Platelet populations were gated on cell size using forward scatter (FSC) and side scatter (SSC) and CD61 positivity, or by FSC and SSC alone in hamster. Light scatter and fluorescence channels were set at logarithmic gain and measurement of the platelet population gate was stopped after 20.000 events per sample or after 180 seconds in case of low platelet counts (thrombocytopenia).

### Statistical Analysis and Data Presentation

Data are presented as mean ± SEM. Statistical analysis was performed by one-way ANOVA with pos hoc Tukey, Wilcoxon signed rank test, one-way ANOVA with post hoc least significant difference, One-Sample T-test, or by ANOVA for repeated measures (SPSS 20.0 for Windows), with P<0.05 considered significantly different. Correlations were calculated using Pearson’s correlation. Sigmaplot 12.0 and SPSS 20 were used to produce the graphs shown in this article.

## Results

### Platelet Dynamics during Natural Torpor

**Figure 1:**
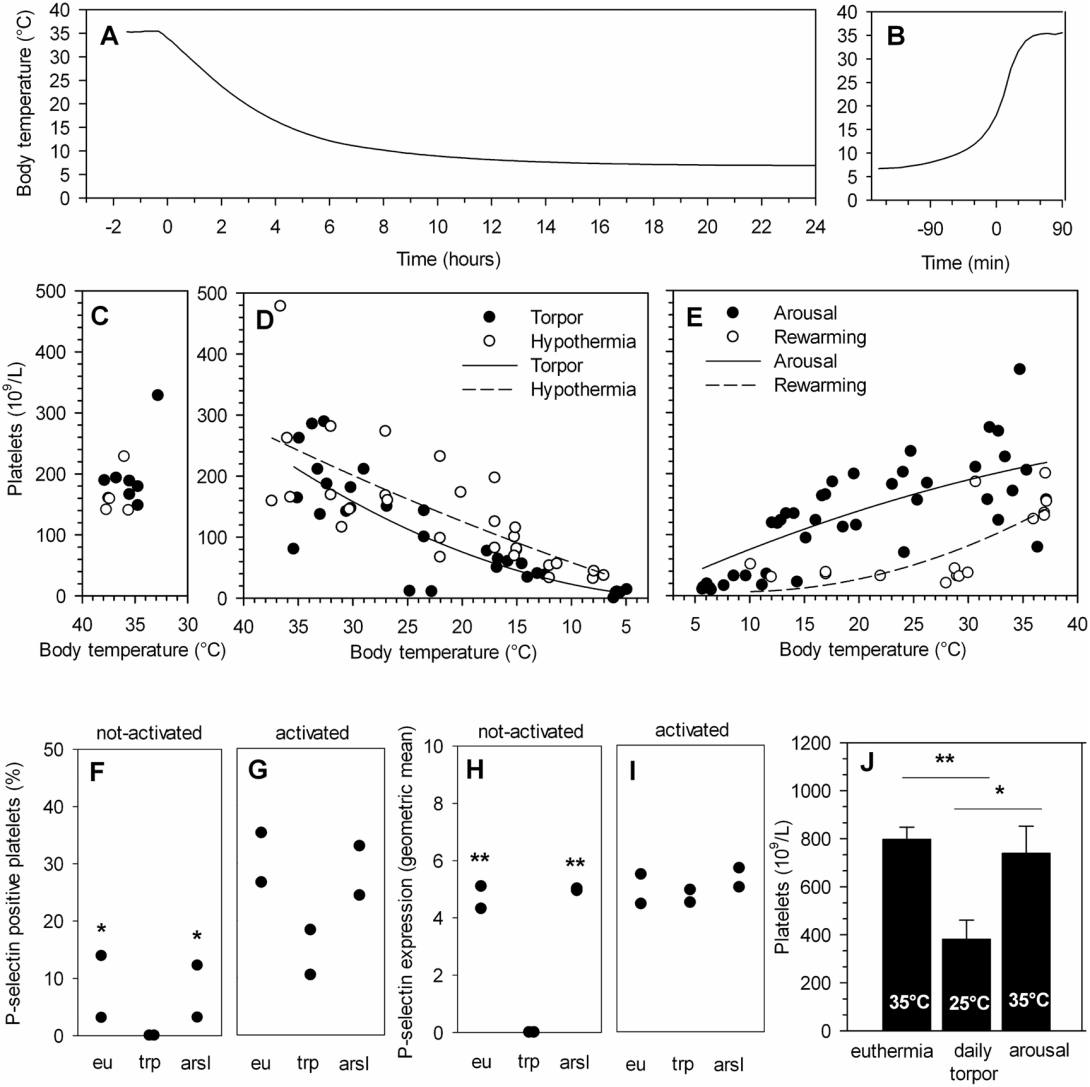
Body temperature dependent platelet count of functional platelets during torpor and arousal in natural hibernating Syrian hamster at 5°C ambient temperature. A) During spontaneous entrance into torpor body temperature gradually declines from 35°C to 8°C in a matter of hours. B) Increase in body temperature during a spontaneous arousal, demonstrating the rapid increase to euthermic level. Line represents one of thirty-one Syrian hamsters, measured with an intraperitoneal implanted Thermochron iButton. C) Normal platelet count in summer-euthermic Syrian hamster (n = 5, open dots; n = 7, black dots). D) Platelet count decreases with lower body temperature from euthermic stage to deep torpor in the Syrian hamster (n = 31), both during natural hibernation as well as during forced hypothermia (n = 8, multiple sampling). Curves from D) and E) are fitted to a polynomial quadratic curve with equation y = y_0_+ax+bx^2^ and constraints of y_0_>0 and y_0_≤ lowest platelet count for torpor. Black dots (•) are natural hibernating hamsters, open dots (°) are forced-cooled hamsters. E) Platelet number increases rapidly to a normal level during arousal (n = 42) or rewarming from forced hypothermia (n = 7, multiple sampling). F) P-selectin positive platelets are absent in torpid hamsters. G) The platelets are activatible following addition of ADP and the subsequent percentage of P-selectin positive platelets is similar to euthermic and aroused animals. H) The P-selectin expression level per platelet was significantly decreased in non-activated platelets from torpor compared to euthermia and arousal hamsters. I) Upon activation with ADP, P-selectin expression reaches similar levels in euthermia (eu), torpor (trp) and arousal (arsl). Please note that F-I are n = 2 per group. J) Circulating platelet count is reduced during daily torpor in the Djungarian hamster, and restored upon arousal. Bars represent mean ± SEM of 5 to 9 animals per group. *P<0.05, **P<0.01.

Platelet count and body temperature were measured during the different phases of hibernation. Body temperature of the Syrian hamster entering torpor decreases from 35°C to 8°C in 12 hours (Figure 1A). In torpor, the number of circulating platelets decreases by 96% from the normal euthermic level of 198×10^9^/L (Figure 1C) to 8×10^9^/L (Figure 1D). The state of torpor lasts for 6–7 days in the Syrian hamster. At the end of torpor, the body temperature increases from 8°C to 35°C during arousal within 180 minutes (Figure 1B). The number of circulating platelets increases in this 3 hour timeframe from 12×10^9^/L to 187×10^9^/L (Figure 1E) approximating the normal euthermic resting rate of 198×10^9^/L (Figure 1C). The platelet count correlates well with body temperature during torpor (Pearson’s R = 0.825; P<0.01, n = 31) and arousal (Pearson’s R = 0.757; P<0.01, n = 42) (Figure 1D–E). Thus, the drop in body temperature during deep torpor in the Syrian hamster is associated with the concurrent thrombocytopenia, and the rise in temperature during arousal associates with a restoration of platelet count.

To assess platelet function throughout hibernation, CD62P expression was determined on platelets in whole blood from Syrian hamsters in euthermia, torpor and arousal (Figure 1F–I). While P-selectin positive platelets are absent in the hamsters in torpor, they are present at normal levels in aroused and euthermic hamster (One-Sample T-test, test value = 0; P<0.05, Figure 1F). In contrast, the percentage of P-selectin positive platelets following activation with ADP of torpid hamster was similar to those of aroused and euthermic animals (Figure 1G). Likewise, the P-selectin expression level of unstimulated platelets was significantly lower in torpid hamster compared to aroused and euthermic groups (One-Sample T-test, test value = 0; P<0.01, Figure 1H). Upon activation with ADP, however, P-selectin expression reaches similar levels in euthermia, torpor and arousal (Figure 1I). Together, these data imply that P-selectin expression on circulating platelets is significantly decreased in torpid hamster, but restores to normal euthermic levels shortly after arousal.

During daily torpor, the body temperature of the Djungarian hamster decreases from 35°C to 25°C. As seen in Figure 1J, the number of circulating platelets is reduced by 52% from euthermic 797×10^9^/L to 381×10^9^/L (P<0.01) during this torpor bout and is restored to 739×10^9^/L (93% of euthermic condition) during arousal with 35°C body temperature (P<0.05; compared to torpor)). Thus, daily torpor in the Djungarian hamster also leads to thrombocytopenia, but to a lesser extent than the deep torpor in Syrian hamster, and platelet count also rapidly restores towards euthermic level upon arousal.

### Forced Hypothermia Induces Thrombocytopenia in Hibernating and Non-hibernating Animals, but Maintains Platelet Function

In order to determine the effect of body temperature on the platelet count irrespective of metabolic suppression during natural torpor, forced hypothermia was induced in anesthetized euthermic (summer-active) Syrian hamsters until a body temperature of 8.7±2.2°C was reached (Figure 1C–E, open dots). Platelet numbers were measured during the process of cooling and rewarming similar to measurements in hibernating Syrian hamster. Platelet count diminishes by forced hypothermia to 78×10^9^/L (Figure 1D, n = 5), a drop of 53% compared to euthermic platelet counts of 166×10^9^/L (Figure 1C, n = 5; Wilcoxon signed rank test, P<0.05), and restores upon rewarming to 149×10^9^/L (Figure 1E, n = 5; Wilcoxon signed rank test, P<0.05) in a similar fashion as during torpor. Additionally, the number of circulating platelets correlated with body temperature during cooling (Figure 1D; Pearson’s R = 0.727; P<0.01, n = 29) and during rewarming following forced hypothermia (Figure 1E; Pearson’s R = 0.660; P<0.01, n = 16). Curves in Figure 1D and 1E have been fitted to a polynomial quadratic curve (y = y_0_+ax+bx^2^) with constraints of y_0_>0 and y_0_≤ lowest platelet count for the data points of torpor (y = 4.9e^−16^+0.81x+0.15x^2^), hypothermia (y = 1.3e^−16^+5.48x+0.04x^2^), arousal (y = 3.8e^−15^+8.21x−0.06x^2^), and rewarming (y = 20–3.04x+0.17x^2^). The curves show a steady decline during torpor and forced hypothermia, and steady incline upon arousal, whereas the rewarming curve shows a delayed but progressive incline towards reaching euthermia.

**Figure 2:**
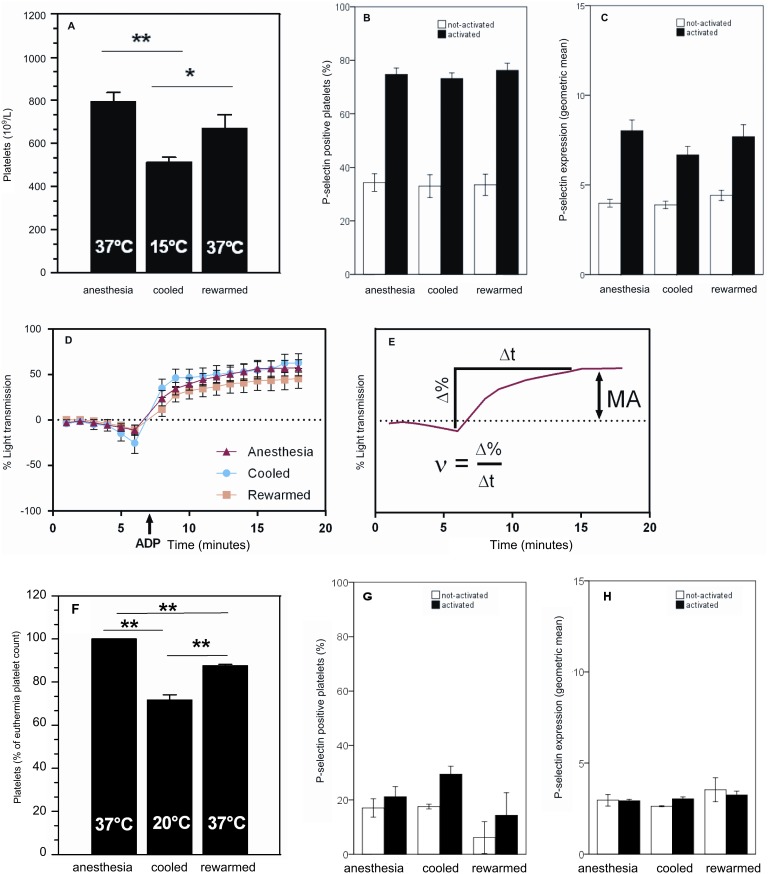
Decreased platelet count with preserved function during forced hypothermia. A) Rats forced to hypothermia of 15°C have a decreased amount of platelets, which partially restores during rewarming. B) No difference in amount of activatable platelets from anestethized euthermic, cooled or rewarmed rats. C) Unchanged P-selectin expression at all time points in both non-activated and activated whole blood samples. D) Unchanged aggregometry at all time points upon addition of ADP. E) Mathematical approach for velocity and max amplitude of platelet aggregation. ν, velocity of aggregation; Δ%, change in percentage light transmission; Δt, timespan over which velocity is determined; MA, maximum aggregation in % light transmission. F) Mice forced to hypothermia of 20°C have a decreased amount of platelets, which partially restores during rewarming. Panels G) and H) show unchanged platelet P-selectin expression between time points in non-activated and activated whole blood samples. Bars represent mean ± SEM of 7 to 27 rats per group and 3 to 9 mice per group. *P<0.05, **P<0.01.

To examine the role of body temperature in a non-hibernator, platelet count and function was assessed in anesthetized rats in which forced hypothermia was induced to reach a body temperature of 15°C. Considering the euthermic number of platelets in rats (793×10^9^/L), circulating platelet count decreases by 35% in the hypothermic condition (513×10^9^/L, P<0.01) and restores upon rewarming to 85% (671×10^9^/L, P<0.05) of euthermic condition (Figure 2A).

To assess platelet function, CD62P expression level and platelet aggregometry was measured on platelets from the forced-cooled rats (Figure 2B–D). The fraction of P-selectin positive platelets does not differ between anesthetized, cooled or rewarmed rats, both in non-activated and ADP activated blood samples (Figure 2B). Furthermore, the P-selectin expression level is similar in platelets from all groups both in non-activated and ADP activated blood samples (Figure 2C). Further, aggregation of rat platelets is unaffected during anesthesia, cooling and subsequent rewarming (Figure 2D). However, while maximum aggregation is similar in all groups, the velocity of aggregation in cooled rats appears to be increased in comparison to anesthetized and rewarmed rats, albeit not reaching a significant difference (Figure 2E and Table 1).

**Table 1:** Aggregation of platelets from forced-cooled rats.

Rat	Velocity (%Light transmission min^−1^)	Max aggregation
**Anesthetized**	12,6±1,82	57,0±8,57
**Cooled**	21,2±5,77	60,5±10,2
**Rewarmed**	12,6±3,70	45,7±11,6

Velocity and max amplitude of aggregation of rat platelets in response to 20 μM of ADP is not significantly different between anesthetized, cooled and rewarmed rats. Values are mean ± SEM of 10 rats per group.

To further corroborate the finding of decreased platelet count upon decreased body temperature, we forced-cooled anesthetized mice (37.2±0.7°C) to a body temperature of 20.1°C±0.3°C for 1 hour and subsequently rewarmed them to 37.5±0.8°C. Platelet count decreases from 1,036×10^9^/L in euthermia to 777×10^9^/L during cooling (28+/−0.02% decrease, P<0.01), and partially restored to 817×10^9^/L (P<0.01, 12+/−0.01% lower than euthermia) upon rewarming (Figure 2F). Thus, platelet counts were significantly lower in 20°C animals compared to rewarmed animals (P<0.01). Forced cooling did not appear of influence on platelet activation, as ADP induced P-selectin expression, was not significantly different between the groups (Figure 2G–H).

Taken together, the reduction in platelet count by forced hypothermia in the rat and mice is less substantial than in the Syrian hamster (35% and 28% versus 53% reduction), while all are less than the reduction during natural deep torpor in the hamster (96%). However, the minimum body temperature reached during forced cooling and natural deep torpor in the Syrian hamster correlates well with platelet numbers, emphasizing the relevance of body temperature in the reduction of platelet numbers (Pearson’s R = 0.727; P<0.01, n = 29 for forced hypothermia hamster and Pearson’s R = 0.825; P<0.01, n = 31 for natural deep torpor hamster).

Further, during forced cooling of rat and mice, platelet function is not altered. This is demonstrated by similar percentages of P-selectin positive platelets from both not-activated or activated blood samples during all timepoints sampled during the cooling/rewarming procedure (Figure 2B). Moreover, the platelets express similar amounts of P-selectin (Figure 2C). Additionally, platelet aggregometry in rat (both velocity and maximum of aggregation) shows no difference between groups (Figure 2D–E, Table 1).

### Platelet Dynamics during Pharmacologically Induced Torpor

**Figure 3:**
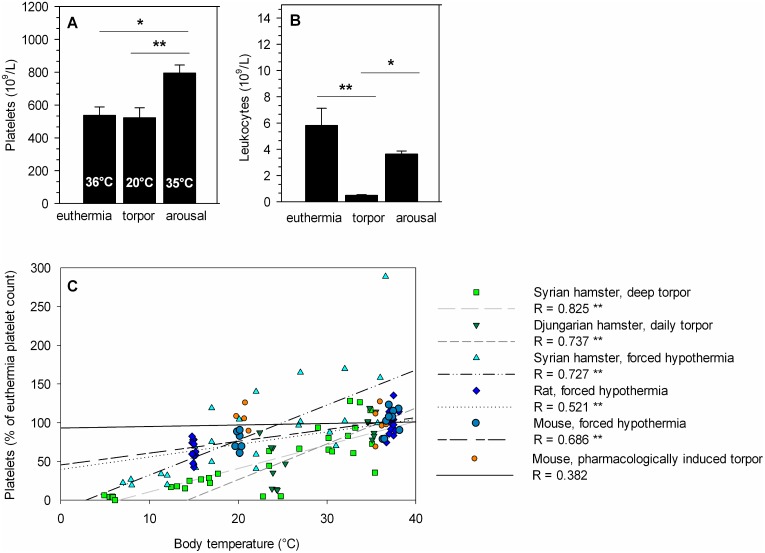
Pharmacologically induced torpor by 5′-AMP does not decrease platelet count despite decreased body temperature. A) Pharmacologically induced torpor by 5′-AMP in mice does not decrease platelet count during torpor and shows an increase upon arousal. Body temperature drops during torpor and restores during arousal. B) Leukocyte level decreases with falling body temperature. C) The correlation of decreased body temperature and reduced platelet count is prominent in deep hibernating hamster (n = 31), daily hibernating hamster (n = 15), forced-cooled hamster (n = 8, multiple sampling), forced-cooled rat (n = 25), and forced-cooled mouse (n = 15), but absent in 5′-AMP induced torpor in mice (n = 10). Bars represent mean ± SEM of 5 to 6 animals per group. *P<0.05, **P<0.01.

5′-AMP can induce a torpor-like state in non-hibernators. This torpor-like state is characterized by a.o. a leukopenia (predominantly lymphopenia) dependent on the decrease in body temperature [32]. To investigate the effect of hypothermia induced by metabolic suppression on platelet count in a non-hibernator, pharmacologic torpor was induced by administrating 5′-AMP to normothermic mice (36.4±0.8°C). Subsequent hypothermia reaches a minimum body temperature of 20.5±0.5°C at 5 hours after injection. At this point the platelet count is similar in 5′-AMP and sham injected animals, amounting 537×10^9^/L versus 523×10^9^/L, respectively (Figure 3A). Platelet count increases when body temperature returns to euthermic value (35.4±0.5°C) after 10 hours and shows a clear elevation, amounting 795×10^9^/L (Figure 3A, P<0.01). Finally, platelet function, as assessed by aggregometry was not changed throughout 5′-AMP induced torpor and arousal in mice compared to sham injected animals. Full irreversible aggregation was observed in all groups (Figure S1 and Table S1). P-selectin positive platelets were present in the same amount in blood samples from torpor and arousal mice compared to euthermia with a similar expression level between the groups (Figure S2–3). To assess whether torpor induction by 5′-AMP was successful, body temperature and leukocyte count were measured, both decreased during torpor [28], [32]. As found previously, body temperature and leukocyte level dropped from 36.4°C and 5.8×10^9^/L in euthermia to 20.5°C (P<0.01) and 0.4×10^9^/L (P<0.01) in torpor, and restored to 35.4°C (P<0.01) and 3.6×10^9^/L (P<0.05) upon arousal respectively (Figure 3B). Thus, while 5′-AMP induced torpor, it does not decrease platelet count or function in mice during torpor, whereas platelet counts are increased upon arousal with increasing body temperature. To further investigate the role of body temperature in the decrease in circulating platelets, all data from the animal experiments were plotted (Figure 3C). The reduced platelet count (expressed as percentage of euthermia platelet count) during cooling or torpor correlates well with decreased body temperature in all animals, except for torpor in mice induced by 5′-AMP administration. The graph shows good correlations for Syrian hamster in deep torpor (Pearson’s R = 0.825; P<0.01, n = 31), Djungarian hamster in daily torpor (Pearson’s R = 0.737; P<0.01, n = 15), forced-cooled Syrian hamster (Pearson’s R = 0.727; P<0.01, n = 29), forced-cooled rat (Pearson’s R = 0.521; P<0.01, n = 26), and forced-cooled mouse (Pearson’s R = 0.686; P<0.01, n = 9). However, there is no good correlation between platelet count and body temperature in 5′-AMP induced torpor in mice (Pearson’s R = 0.382; P>0.05, n = 16).

### Storage and Release as Mechanism of Thrombocytopenia

**Figure 4:**
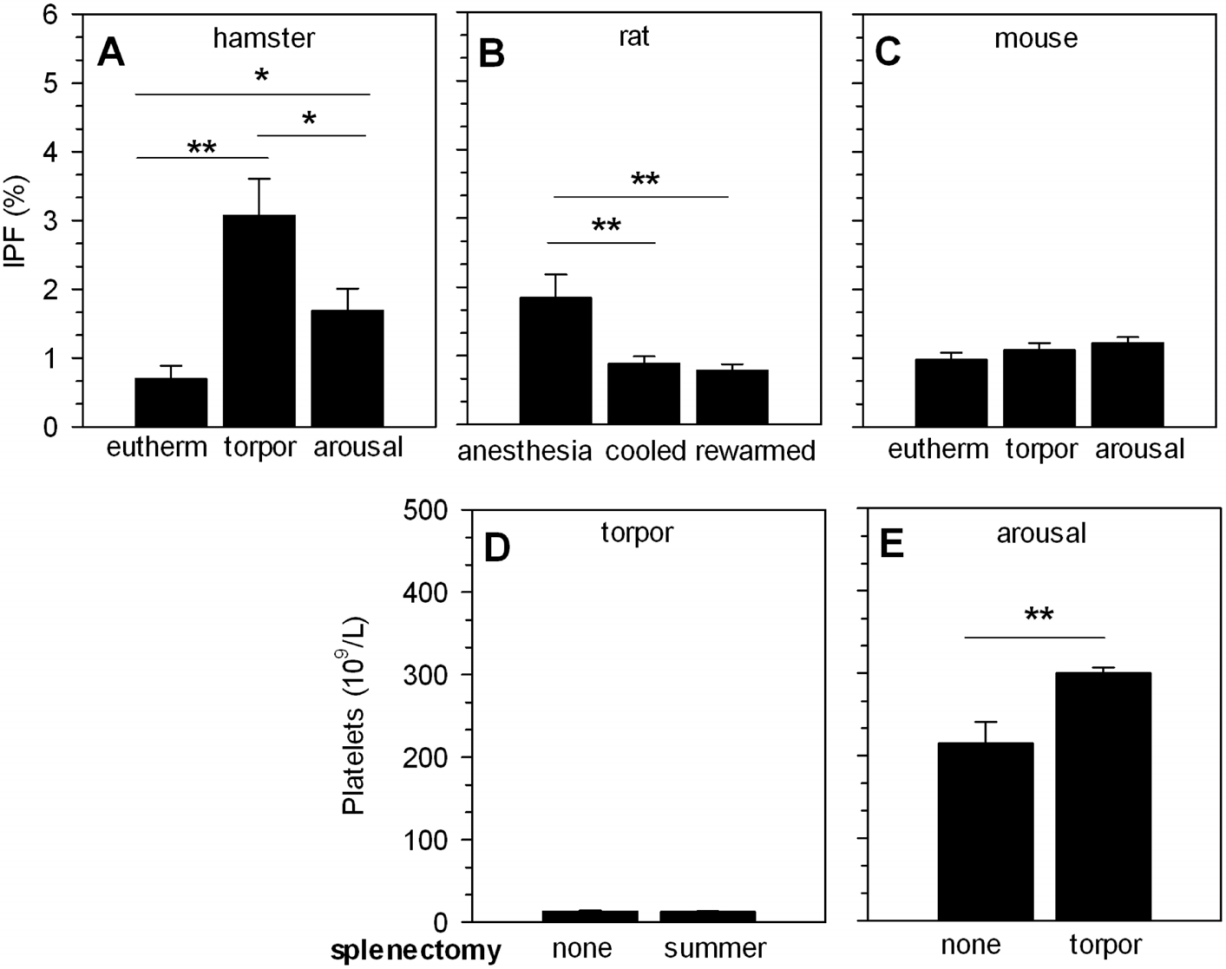
Restoration of circulating platelet numbers during arousal and rewarming does not originate from spleen or bone marrow. A) Immature platelet fraction (IPF) is increased in torpor, but decreases in arousal toward normal euthermic percentage in Syrian hamster. B) In rat, IPF decreases during cooling and rewarming. C) In mice IPF only increases during arousal. D) Splenectomy prior to hibernation does not inhibit induction of thrombocytopenia in torpor. E) Splenectomy during torpor does not prevent restoration of platelet count during the subsequent arousal. Bars represent mean ± SEM of 4 to 12 animals per group. *P<0.05, **P<0.01.

Given the rapid restoration of platelet counts upon arousal or rewarming, our data suggest that thrombocytopenia occurs due to storage-and-release, rather than clearance-and-reproduction. To further establish whether bone marrow massively releases fresh platelets upon arousal or rewarming, we determined the immature platelet fraction (IPF) in peripheral blood of Syrian hamsters after arousal, in forced-cooled rats after rewarming, and in mice after arousal of pharmacological induction of torpor (Figure 4A–C). The IPF increases from 0.7% in euthermic Syrian hamsters to 3.1% during torpor (P<0.01), followed by a decrease to 1.7% upon arousal (P<0.05, Figure 4A). In rats, the IPF reduces from 1.8% during anesthesia to 0.9% during cooling (P<0.01) and 0.8% after rewarming (P<0.01; Figure 4B). The IPF in mice did not change from euthermia to torpor and arousal (0.9%, 1.1%, 1.2% respectively; Figure 4C). Consequently, a massive increase in IPF is absent upon arousal and rewarming, which strongly substantiates the hypothesis that restoration of platelets is caused by release from a storage site.

The spleen is a platelet sequestering organ [33] and can function as a platelet reservoir [34]. To investigate a potential role of spleen in the regulation of circulating platelet numbers during hibernation, splenectomies were performed either before hibernation or during torpor in the Syrian hamster. Splenectomy before the hibernating season did not preclude the induction of thrombocytopenia during torpor (Figure 4D), suggesting the spleen is not needed to sequester platelets in this phase. To investigate the opposite, i.e. whether the spleen is involved in restoration of platelet counts during arousal, splenectomy was performed during torpor. In this case, splenectomy did not impede the restoration of platelet count during arousal compared to arousal with native spleen (300×10^9^/L vs. 215×10^9^/L, P<0.01, respectively; Figure 4E). Thus, these experiments demonstrate that the spleen is neither essential for platelet storage during torpor, nor for restoration of platelet count upon arousal.

## Discussion

In the current study we demonstrate that thrombocytopenia as observed in deep and daily torpor is not confined to hibernating animals. Also non-hibernators decrease their platelet count during forced hypothermia. The thrombocytopenia in both hibernators and non-hibernators is reversible upon arousal and rewarming, respectively. Thus, this study suggests that body temperature is a main driving factor for thrombocytopenia during hibernation. Moreover, this study suggests that platelet intrinsic function is maintained throughout torpor/arousal in hibernators as well as throughout cooling/rewarming and pharmacological induced torpor, as demonstrated by P-selectin expression and platelet aggregometry. Importantly, however, in natural torpor, circulating platelets were found not to express P-selectin in contrast to force-cooled rat, mouse, and 5′-AMP injected mouse. Finally, aggregometry indicates that neither the velocity nor the maximum aggregation show any changes among groups of forced-cooled rats and pharmacological torpor in mice.

Further, the decrease in body temperature during the initiation and continuation of deep torpor and during forced hypothermia in the Syrian hamster correlates well with the reduction in platelet count in peripheral blood. Moreover, thrombocytopenia is present in both deep and daily torpor, which demonstrates that this phenomenon is not confined to deep torpor only. Furthermore, forced hypothermia induces thrombocytopenia both in hibernators and non-hibernators, i.e. hamster, rat and mouse. The decrease in body temperature correlates well with the decrease in platelet count in deep and daily torpor, and forced hypothermia in both hamster, rat and mouse. These correlations suggest a similar underlying mechanism of temperature dependent platelet dynamics in both hibernating and non-hibernating mammals. Likewise, in both deep and daily hibernating hamsters and in forced-cooled rats and mice, platelet count increased rapidly to euthermic level upon arousal and rewarming. The more rapid recovery of the platelet count in hamsters aroused from natural torpor as compared to forced-cooled hamster may be caused by a hysteresis effect of core body temperature increase. Whereas, during arousal the body temperature is increased from the inside out, the body temperature following forced-cooling increases from the outside in. Ultimately, this may result in a slower warming of platelet storage sites in forced-cooled animals. Interestingly, while *ex vivo* cooling initiates the rapid clearance of platelets by the liver upon reinfusion in mice and humans [35], [36], such effect may be absent following *in vivo* cooling, as in our study hamster, mouse and rat platelet numbers were restored upon rewarming. However, it is difficult to compare *in vivo* observations to *ex vivo* experiments, especially when accounting for the fact that the extend of cooling of mice and rats to 20°C and 15° respectively is markedly less profound than 4°C *ex vivo* storage.

In contrast, the platelet count is unaffected during pharmacological induced torpor in mice by 5′-AMP despite the decrease in body temperature. Moreover, upon arousal the platelet count surpasses the initial euthermic level when body temperature returns to normal, suggesting a release of already stored platelets, compensating the platelet reduction during torpor induced by decreasing body temperature. Most likely, the different pattern in change of platelet count in 5′-AMP induced torpor compared to natural hibernation and forced cooling is attributable to the effect of the compound on platelet function (see also below) [37].

Contrarily to immature platelet levels in hibernating ground squirrel [22], there was a relative, but marginal, increase in immature platelet fraction (IPF) during torpor of Syrian hamster, but not in cooled rat or induced torpor in mouse. The small increase in IPF, however, cannot account for the massive increase of platelet count upon arousal. Increased IPF in torpid hamster may result from a decreased clearance of immature platelets during torpor compared to mature platelets. Further, the difference in IPF between torpid squirrel and hamster may reflect a species difference. Alternatively, the method used to determine IPF in these studies differs, which may well result in the difference in IPF count between species. In addition, whether hamster platelets change their shape upon cooling, as described in ground squirrel (22), is not yet known. Possibly, the shape change influences the flow cytometric measurement of platelets. The latter seems less likely an explanation, as samples were stored at room temperature before processing, allowing for reversal of the potential shape change (22), thus granting normal platelet counts during flow cytometry. Further, in all animal species, the rapid restoration of platelet count in face of the marginal changes in the IPF support a storage-and-release mechanism over a clearance-and-reproduction mechanism to underlie thrombocytopenia of torpor and forced cooling. Finally, by splenectomizing animals we revealed that the spleen is not crucial to either induce or restore thrombocytopenia during natural hibernation. Taken together, while in 5′-AMP induced torpor thrombocytopenia is not present, inhibition of coagulation during natural torpor and forced cooling is instituted by a body temperature dependent reduction in the number of circulating platelets, rather than on the inhibition of their function.

Our data suggest that low body temperature induces clearance of free circulating cells, likely by storage-and-release. Release of newly formed platelets from the bone marrow is unlikely to play a significant role in the restoration of normal platelet counts upon rewarming, even though the steady state megakaryocytopoiesis supplies 10^11^ platelets daily and can increase 10-fold on demand [27]. Supporting this, the immature IPF was not significantly increased upon arousal in our study. Therefore, the rapid and full restoration of platelet count during arousal is unlikely to result from release of newly formed platelets from the bone marrow. Likely, storage of platelets governs thrombocytopenia during torpor. A potential storage location might be the spleen [26], which has a relatively large capacity to sequester and destroy (abnormal) platelets as compared to other organs [33]. Moreover, the spleen can release platelets into the circulation after sequestration [34]. In hibernating ground squirrel, potential platelet storage sites include spleen, but also lungs and liver as all three appeared to sequester platelets [26]. However, by splenectomizing animals before torpor, we revealed that the spleen does not play an essential role in the induction of thrombocytopenia. Further, by splenectomizing animals during torpor we demonstrate that the spleen neither plays a key role in the restoration of normal platelet counts, as splenectomy did not prevent the restoration of normal platelet counts upon arousal. Although these results suggest no essential role for the spleen in the induction of thrombocytopenia or restoration of normal platelet counts, a potential role cannot be ruled out. In a study from the 1970’s, Reddick et al. reported that thrombocytopenia was precluded by splenectomy prior to hibernation in the 13-lined ground squirrel [26]. While details of splenectomy are not included and splenectomy did not increase platelet count in non-hibernating squirrels, it is difficult to comment on possible causes for the apparent different observations. As this is possibly due to species differences, future studies taking a similar approach as ours with respect to timing of splenectomy are needed to confirm this assumption. Notably, splenectomy during torpor increased the amount of platelets upon arousal. Thus, our findings imply a role for spleen in the sequestration of platelets during arousal rather than in their release. The alternative would be that spleen is essential to sequester and release platelets in hibernation, but that the effects of splenectomy are masked by the effects of abdominal surgery. This would imply that surgery in summer induces sequestration of platelets in organs other than spleen months later, which appears less likely. Further, surgery during torpor may induce release of platelets from other sources than spleen (reactive thrombocytosis). However, as these platelets are mostly bone-marrow derived, this normally leads to a large increase in IPF, which is absent in our animals. Thus, the most likely explanation is that spleen does not play an essential role in platelet dynamics during torpor.

In euthermic conditions part of the platelet count is sequestered in the spleen [33], [38]. After splenectomy, this sequestering capacity will be decreased. Thus, the reversible hypothermia induced thrombocytopenia and low IPF during torpor and arousal advocate a storage-and-release mechanism of platelets. The spleen, as natural platelet sequestering organ, is not essential for potential platelet storage during torpor, nor for restoration of platelet count upon arousal, but might play a role in platelet sequestration during arousal.

By splenectomizing animals, we demonstrated that the spleen does not play a key role in the induction of thrombocytopenia during torpor. Platelets can reversibly adhere to arterioles, venules, and capillaries by means of margination. Therefore, a potential storage mechanism of platelets during torpor might be platelet margination. By computational and experimental methods several factors promoting platelet margination are revealed, including increased hematocrit [39], platelet shape (spherical particles marginate more quickly) [40], lower flow rate [41], and augmented expression of adhesion molecules [42]. Of these factors, hematocrit is increased [24], flow rate is decreased [15], and platelet shape changed to spherical at a body temperature<25°C during torpor in 13-lined ground squirrels [26]. Changes in platelet shape are mediated by intracellular cytoskeletal microtubule rearrangements and are reversible upon rewarming in 13-lined ground squirrel platelets [22], and partly reversible in mice and humans [35], [43], [44]. Therefore, reversible platelet shape change might contribute to the storage-and-release mechanism mediated by platelet margination.

Besides changes in hemodynamics and platelet shape, increased adhesion molecule expression might promote platelet margination during torpor as well. In this perspective, our observation that P-selectin expression is absent on circulating platelets during torpor in the Syrian hamster is intriguing. One of the attractive hypotheses is that hibernating animals indeed increase P-selectin expression on platelets upon entrance into torpor to induce margination. Consequently, only a small fraction of platelets not expressing P-selectin remains in the circulation. Nevertheless, the remainder of circulating platelets from torpid animals can still be activated, possibly to ensure appropriate coagulation during arousal should this be needed. However, future studies should address P-selectin expression and of other adhesion molecules, such as ICAM-1, alphavbeta3 integrin and GPIbalpha [45], [46], in hibernating animals in more detail.

One of the factors that might add to increased margination of platelets in torpor or cooling is hypoxia. Hypoxia during torpor might lead to exocytosis of endothelial cell Weibel-Palade bodies and subsequent release of von Willebrand factor and P-selectin expression [47], both stimulating platelet binding to the endothelial cell. Also, during torpor, the endothelial adhesion molecules VCAM-1 and ICAM-1 are modestly upregulated in the lungs, followed by normalization during arousal [14]. Further, *in vitro* experiments reveal that plasma from hibernating, but not summer euthermic ground squirrels, stimulates the expression of ICAM-1, VCAM and E-selectin on rat endothelial cells [48]. Together, endothelial activation leading to the expression of adhesion molecules may stimulate platelet margination in torpid animals.

Despite the procoagulant state of torpor due to low blood flow [15], increased blood viscosity [16], [17], immobility, chronic hypoxia, and low body temperature [5], no organ injury has been demonstrated after arousal [5], advocating absence of thromboembolism during hibernation. We speculate that margination of platelets might prevent thromboembolism formation during torpor. Taken together, margination-promoting factors during torpor might well underlie the clearance of free circulating functional platelets shown in this study upon lowering of body temperature.

While a decrease in platelet count was observed in hibernators and forced-cooled animals, pharmacological induction of torpor by 5′-AMP did not induce thrombocytopenia, despite clear reductions in body temperature and leukocyte count. The most likely explanation for this discrepancy is that 5′-AMP interferes with the temperature dependent regulation of platelet counts. Interestingly, very recently it has been shown that 5′-AMP inhibits platelet function, including the inhibition of P-selectin expression upon platelet activation [37]. In the same study the adenosine A_2A_ receptor is activated by 5′-AMP and inhibits platelet function. A_2A_ receptor is not the main target of 5′-AMP for the induction of torpor. Induced torpor acts via A1 receptors in Syrian hamster, ground squirrel and rat [49]–[51]. 5′-AMP has been shown to be a true A1 receptor agonist [52]. These studies, however, did not measure platelet count. Likely, stimulation of A1 receptor alone is not sufficient to induce thrombocytopenia in mice. Our finding that P-selectin is absent in circulating platelets of torpid animals and that 5′-AMP inhibits platelet function may thus implicate that platelet functionality, particularly the expression of adhesion molecules, is essential for the temperature dependent decrease in platelet count in torpor and cooling.

While our data demonstrate platelet aggregation not to be affected largely by torpor or cooling, some technical limitations may apply. For flow cytometry analyses 10 uM ADP was used as platelet agonist, effective for hamster and rat platelets, but elicited only minimal activation in mouse platelets. Studies are ambiguous if ADP sensitivity is sex dependent, potentially C57BL/6J male mice are less sensitive to platelet agonists than female littermates [53]. Due to limitations in sample volume of rodent blood, platelet aggregation was determined with a microtiterplate assay (MTP) rather than the classical light transmittance aggregometry (LTA). While optimal platelet concentrations of 600×10^9^/L for MTP have been reported [31], platelet yields did not allow for an equal platelet concentration among each experiment. To compensate for these differences in platelet concentrations, aggregation of mouse and rat platelets was compared to an internal standard which was matched in platelet concentrations, which allowed representation of the data as a percentage of the internal standard. Moreover, the MTP method has been reported to have a lower sensitivity than LTA when low concentrations of agonist are used [54]. However, this difference in sensitivity is deemed absent at higher agonist concentrations, motivating the use of 20 μM ADP to induce a full irreversible aggregation. Finally, since aggregation of rodent platelets is measured in a buffer instead of in plasma, the effect of any plasma factor that influences platelet aggregation may be lost, e.g. a decrease in coagulation factors as seen in the 13-lined ground squirrel [22].

Up to now, human platelets intended for transfusion are stored up to 5 days on room temperature, risking bacterial contamination [54], because cold storage on the other hand leads to aggregation upon rewarming and other detrimental effects that change platelet function [55], [56]. Finding ways for cold storage of platelets, a.o. to prevent bacterial growth, while preserving platelet function, would reduce transfusion associated infections and lead to increased use before expiration date. This study introduces the possibility of a shared mechanism between non-hibernating and hibernating mammals for reversible hypothermia induced hypocoagulability, via platelet storage in the cold with preserved platelet function.

## Conclusion

During torpor, free circulating platelets are cleared from the blood. The resulting thrombocytopenia is reversible and due to a lowering in body temperature. The hypothermia induced thrombocytopenia is not confined to deep torpor or hibernating animals, as it was also observed in daily torpor and upon forced hypothermia in non-hibernators. Decreased platelet count does not coincide with decreased platelet function, and recovers rapidly upon arousal and rewarming due to release of retained platelets. Platelet storage and release in hibernators are not mediated by the spleen. Understanding the underlying mechanisms that govern the reversible hypothermia induced thrombocytopenia, with preservation of platelet function, might yield improved uses for therapeutic hypothermia, as well as potential cold storage of human platelets, extending their shelf life.

## Supporting Information

Figure S1 **Platelet aggregation of mouse platelet suspensions does not differ between euthermia and pharmacologically induced torpor and arousal.** ν, velocity of aggregation; Δ%, change in percentage light transmission; Δt, timespan over which velocity is determined; MA, maximum aggregation in % light transmission. Data is shown as the mean (n = 6 euthermia, n = 5 torpor, n = 7 arousal) ±SEM.
(TIF)

Figure S2 **Normal platelet activation during pharmacologically induced torpor and arousal.** No difference in amount of activatable platelets from euthermic, torpid or aroused mice. Bars represent the mean (n = 6 euthermia, n = 5 torpor, n = 7 arousal) ±SEM.
(TIF)

Figure S3 **Similar P-selectin expression on platelets during euthermia and pharmacologically induced torpor and arousal.** Unchanged P-selectin expression at all time points in both non-activated and activated whole blood samples. Bars represent the mean (n = 6 euthermia, n = 5 torpor, n = 7 arousal) ±SEM.
(TIF)

Table S1 **Maintenance of velocity and maximum amplitude of platelet aggregation in pharmacologically induced torpor in mice.** Velocity is the slope of % light transmission per minute in the first 5 minutes after addition of agonist. Max amplitude is the mean light transmission of the last three measurements when a stable plateau is observed. One-way ANOVA showed no significant differences between groups (P>0.05). Data is shown as mean (n = 6 euthermia, n = 5 torpor, n = 7 arousal) ± SEM.
(DOCX)
