# Supplementary figures and images for: Platelet Dynamics during Natural and Pharmacologically Induced Torpor and Forced Hypothermia
Source: PLoS One. 2014 Apr 10;9(4):e93218. doi: 10.1371/journal.pone.0093218 (PMC3982955; doi:10.1371/journal.pone.0093218)

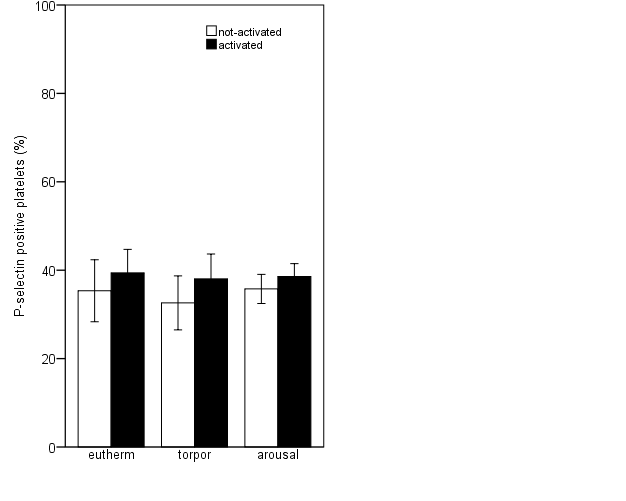

Supplement: Figure S2 — Normal platelet activation during pharmacologically induced torpor and arousal. No difference in amount of activatable platelets from euthermic, torpid or aroused mice. Bars represent the mean (n = 6 euthermia, n = 5 torpor, n = 7 arousal) ±SEM. (TIF) [file pone.0093218.s002.tif]

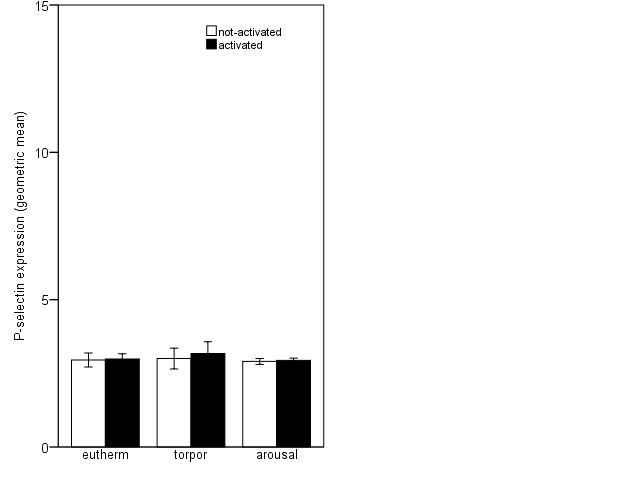

Supplement: Figure S3 — Similar P-selectin expression on platelets during euthermia and pharmacologically induced torpor and arousal. Unchanged P-selectin expression at all time points in both non-activated and activated whole blood samples. Bars represent the mean (n = 6 euthermia, n = 5 torpor, n = 7 arousal) ±SEM. (TIF) [file pone.0093218.s003.tif]
