# Supplementary material for: Platelet Dynamics during Natural and Pharmacologically Induced Torpor and Forced Hypothermia
Source: PLoS One. 2014 Apr 10;9(4):e93218. doi: 10.1371/journal.pone.0093218 (PMC3982955; doi:10.1371/journal.pone.0093218)
Supplement: Table S1 — Maintenance of velocity and maximum amplitude of platelet aggregation in pharmacologically induced torpor in mice. Velocity is the slope of % light transmission per minute in the first 5 minutes after addition of agonist. Max amplitude is the mean light transmission of the last three measurements when a stable plateau is observed. One-way ANOVA showed no significant differences between groups (P>0.05). Data is shown as mean (n = 6 euthermia, n = 5 torpor, n = 7 arousal) ± SEM. (DOCX) [file pone.0093218.s004.docx]

Table S1. Velocity and max amplitude of mouse platelet aggregation in response to 20 µM ADP

| Mouse | Velocity (%Light transmission min^-1^) | Max amplitude |
| --- | --- | --- |
| Euthermia | 11,7 ± 4.86 | 32,6 ± 15.7 |
| Torpor | 9,56 ± 4.40 | 30,4 ± 8.9 |
| Arousal | 12,2 ± 3.49 | 31,6 ± 8.4 |

Velocity and max amplitude of aggregation of mouse platelets in response to 20 µM of ADP is not significantly different in all euthermic, torpid and aroused mice. Values are mean (n=6 euthermia, n= 5 torpor, n=7 arousal) ± SEM.
